# Chromatin remodeling factor LSH affects fumarate hydratase as a cancer driver

**DOI:** 10.1186/s40880-016-0138-7

**Published:** 2016-07-30

**Authors:** Shuang Liu, Yong-Guang Tao

**Affiliations:** 1 Center for Medicine Research, Xiangya Hospital, Central South University, Changsha, 410008 Hunan P. R. China; 2 Cancer Research Institute, School of Basic Medicine, Central South University, Changsha, 410078 Hunan P. R. China; 3 Key Laboratory of Carcinogenesis and Cancer Invasion (Central South University), Ministry of Education, Changsha, 410078 Hunan P. R. China

**Keywords:** Lymphocyte-specific helicase (LSH), Epithelial-mesenchymal transition (EMT), E-Cadherin, ZO-1, Vimentin, Tricarboxylic acid cycle intermediates, Inhibitor of nuclear factor kappa-B kinase alpha (IKKα), G9a

## Abstract

Cancer metabolism and epigenetic alteration are two critical mechanisms for tumorigenesis and cancer progression; however, the dynamic interplay between them remains poorly understood. As reported in the article entitled “Chromatin remodeling factor LSH drives cancer progression by suppressing the activity of fumarate hydratase,” which was recently published in *Cancer Research*, our group examined the physiological role of lymphocyte-specific helicase (LSH) in nasopharyngeal carcinoma (NPC) by focusing on cancer progression and the tricarboxylic acid cycle. We found that LSH was overexpressed in NPC, and its expression associated with Epstein-Barr virus infection. We also found that LSH directly suppressed fumarate hydratase (FH), a key component of the tricarboxylic acid cycle, in combination with euchromatic histone-lysine N-methyltransferase 2 (EHMT2), also known as G9a. Depletion of FH promoted epithelial-mesenchymal transition (EMT). Moreover, LSH controlled expression of tricarboxylic acid cycle intermediates that promote cancer progression, including EMT, through activation by inhibitor of nuclear factor kappa-B kinase alpha (IKKα), a chromatin modifier and transcriptional activator. Our study showed that LSH plays a critical role in cancer progression, which has important implications for the development of novel strategies to treat NPC.

## Background

Cancer metabolism and epigenetic alteration, especially in chromatin remodeling, are two critical mechanisms for tumorigenesis and cancer progression; however, the dynamic interplay between them in tumors remains poorly understood [1–3]. Lymphocyte-specific helicase (LSH), also called helicase, lymphoid-specific (HELLS), is a member of the ATP-dependent helicase in sucrose nonfermenting 2 (SNF2). LSH is not only involved in DNA methylation, but it also promotes PolII stalling or transcriptional pausing [3–5]. We confirmed that LSH interaction with long non-coding RNA (LncRNA) HOX transcript antisense RNA (HOTAIR) regulates the ratio of FoxA1 to FoxA2 and plays a critical role in lung cancer [6]. Epithelial-mesenchymal transition (EMT) is thought to be activated in cancer cells, linked to their dissociation from the primary tumor and their intravasation into blood vessels [7]. However, the effect of EMT in cancer progression, especially in chromatin remodeling, remains poorly understood. But now, based on the He et al. study, “Chromatin remodeling factor LSH drives cancer progression by suppressing the activity of fumarate hydratase,” published in *Cancer Research* [8], the interplay between epigenetic controls in chromatin remodeling and EMT has been addressed.

Oncoprotein latent membrane protein 1 (LMP1) encoded by Epstein-Barr virus (EBV) infects more than 90% of the global adult population and contributes to several malignancies, including nasopharyngeal carcinoma (NPC) [9–11], which is a common cancer in South China and in Southeast Asia [12, 13]. Epigenetic changes induced by EBV and its products, such as LMP1, are key events in the viral process of carcinogenesis, including transcriptional pausing [10, 14]. Chromatin remodeling factors are crucial factors of epigenetics and play a critical role in the development of several malignancies [15], but their role in the progress of NPC remains unknown. Recent work from our group provides robust evidence that LSH is highly expressed in NPC, where it is controlled by LMP1 [8]. Furthermore, our group found that LSH not only promotes growth, migration, and invasion of NPC cancer cells in vitro but also links with EMT, including cell migration, invasion, and tumor growth and colonization in vivo [8], suggesting that LSH plays a critical role in tumor growth and metastasis. Based on these findings, it is clear that LSH promotes transition from the epithelial stage to the mesenchymal stage.

Altered cellular metabolism, in particular the Warburg effect, is a hallmark of cancer cells, with the tricarboxylic acid (TCA) cycle at the center of oxidative metabolism, serving as a robust source for intermediates that are required for anabolic reactions [16]. The mitochondrial enzyme fumarate hydratase (FH), a key component of the TCA cycle, catalyzes the hydration of fumarate to malate and is essential for cellular energy production and macromolecular biosynthesis. In studying the molecular mechanism and seeking to identify potential targets mediating the TCA cycle, our group found (using polymerase chain reaction array analysis) a repressive regulatory role of LSH in FH expression. We also confirmed that LSH is an important regulator of FH expression and down-regulates FH protein level in NPC derived from xenograft and clinical samples. We found that LSH was associated with the *fh* promoter; therefore, FH may serve as a direct target of LSH function. However, LSH may repress the *fh* promoter independent of DNA methylation, even though LSH is linked with DNA methylation, indicating that another mechanism is involved. G9a, also known as euchromatic histone-lysine N-methyltransferase 2, is an important epigenetic regulator, which monomethylates and dimethylates lysine-9 [17]. Our group provided the evidence of an interaction between LSH and G9a; the evidence of recruitment of G9a to the *fh* promoter in a LSH-dependent manner; and the evidence of subsequent chromatin modification leading to FH promoter repression [8], thus linking epigenetic regulation by LSH with suppression of the emerging tumor suppressor gene *FH*.

Oncometabolites are metabolites whose abnormal expression causes metabolic and epigenetic dysregulation and transformation to malignancy [18]. Inactivation of genetic mutations alters the level of 2-oxoglutarate-dependent oxygenases and leads to epigenetic deregulation of oncogenes or tumor suppressors [18, 19]. Diverse metabolites serve as cofactors or substrates for enzymes involved in the deposition or exchange of epigenetic marks, initiating a metabolite-driven pathway of gene regulation [20]. α-Ketoglutaric acid (α-KG) has been shown to influence the pluripotency state in embryonic stem cells via alternation of multiple chromatin modifications [21]. In our study, we found that TCA cycle intermediates and the ratio of α-KG/succinate and α-KG/fumarate are regulated by LSH [8]. However, we found no association between the EBV status and the intermediates of TCA cycles in NPC patients.

The functional interactions between EMT-inducing transcription factors and the modulators of chromatin configuration are central to the underlying mechanism of cancer progression [22]. Metabolic competition can drive cancer progression [23], and this competition is due to the disturbed balance of TCA intermediates that can trigger EMT. The reprogramming of gene expression during EMT is initiated and controlled by signaling pathways that respond to extracellular cues and lead to metabolic reprogramming. In our study, we showed that overexpression of LSH is linked to EMT by increasing migration and invasion ability in NPC [8]. This finding also indicated that EMT induction by LMP1 is mediated by LSH. Furthermore, we found that many other key regulators that induce EMT, such as TWIST and Snail, are affected by LMP1. Moreover, TCA intermediates promote cancer progression through the decrease of epithelial markers and the increase of mesenchymal marker expression.

Recently, abnormal levels of TCA intermediates were shown to activate nuclear factor kappa-B (NF-κB) using a non-canonical pathway independent of inhibitor of nuclear factor kappa-B kinase alpha (IKKα) [24]. Depending on the type of malignancy, IKKα can provide both tumor-promoting and tumor-suppressive mechanisms that are, in most instances, cell autonomous. We recently found that IKKα is diversely expressed in keratinizing and non-keratinizing carcinomas even in the same type of cancer [25]. In addition, we showed that IKKα can localize to the nucleus and that nuclear IKKα can directly bind to the promoters of inflammation factors and leucine-rich repeat containing G-protein-coupled receptor 5 (LGR5), a stem cell marker, thereby up-regulating LGR5 expression through activation of signal transducer and activator of transcription 3 (STAT3) signaling pathway during cancer progression [25, 26]. The chromatin regulator and transcriptional activator IKKα may be involved in the regulation of EMT markers, mediating the effect of LSH and TCA intermediates. LSH overexpression, as well as de-regulation of TCA intermediates, leads to IKKα recruitment to the promoters of EMT-related genes. In this way, LSH induces a cascade of epigenetic and metabolic changes that result in further epigenetic regulations via IKKα and EMT.

**Fig. 1:**
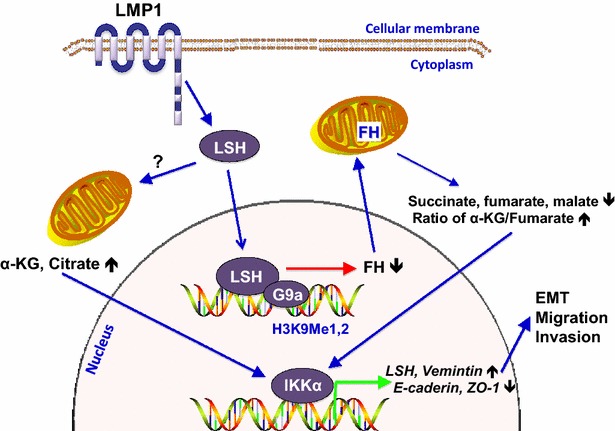
Schematic model of lymphocyte-specific helicase (LSH) in cancer progression. LSH promotes cell growth, migration, and invasion, which are characteristics of cancer progression. The effect of LSH is, in part, mediated by fumarate hydratase (FH), through the intact combination of LSH and euchromatic histone-lysine N-methyltransferase 2 (G9a). FH repression, in turn, leads to changes of tricarboxylic acid cycle (TCA) intermediates, including succinate, fumarate, and malate, and an increase in the ratio of α-ketoglutarate (α-KG) to fumarate. TCA intermediates promote migration, invasion, and epithelial-mesenchymal transition (EMT) through the decrease of E-cadherin and tight junction protein ZO-1 and the increase of vimentin. The changes of E-cadherin and ZO-1 are mediated by inhibitor of nuclear factor kappa-B kinase alpha (IKKα), which directly binds to these promoters

Based on our findings, we propose a model for LSH-mediated signaling and enhancement of NPC tumorigenesis (Fig. 1). In this model, LSH acts as a driver of cancer progression, involving EMT, invasion, and migration. Specifically, LSH, together with G9a, represses FH. Reduced FH level leads to a reduction of succinate, fumarate, and malate; it also increases the ratio of α-KG to fumarate. TCA intermediates, including α-KG and citrate, decrease E-cadherin and ZO-1 expression and increase vimentin expression. The changes of EMT marker gene expression are controlled by IKKα, which binds directly to these promoters as a chromatin bona fide modifier. The pathway leads to EMT, promoting migration, invasion, and cancer progression. However, several issues still need to be addressed. First, it remains unclear which signaling pathway is involved in the regulation of LMP1 on LSH (LMP1 is a membrane protein and LSH is a chromatin remodeling in the nucleus). Second, it is necessary to address how and why, in tumor tissues from patients, TCA intermediates change in high levels of LSH expression.

## Conclusions

Our findings suggest that LSH acts as a driver in NPC by promoting EMT, cell growth, migration, and invasion, which are key characteristics of cancer progression. Our study highlights the importance of LSH-mediated regulation of TCA intermediates in cancer progression. Repression of LSH and its downstream effects may serve as a potential target for novel therapeutic strategies.

## Abbreviation

LMP1: latent membrane protein 1
