# Erratum to: Chromatin remodeling factor LSH affects fumarate hydratase as a cancer driver

**DOI:** 10.1186/s40880-016-0156-5

**Published:** 2016-11-28

**Authors:** Shuang Liu, Yong-Guang Tao

**Affiliations:** 1 Center for Medicine Research, Xiangya Hospital, Central South University, Changsha, 410008 Hunan P. R. China; 2 Cancer Research Institute, School of Basic Medicine, Central South University, Changsha, 410078 Hunan P. R. China; 3 Key Laboratory of Carcinogenesis and Cancer Invasion (Central South University), Ministry of Education, Changsha, 410078 Hunan P. R. China

## Erratum to: Chin J Cancer (2016) 35:72 DOI 10.1186/s40880-016-0138-7

After publication of this article [1], the editor noticed two errors in Figure 1. The word “Vemintin” should be “Vimentin” and “E-caderin” should be “E-cadherin”. The correct version of Fig. 1 can be found in this erratum.

**Fig. 1:**
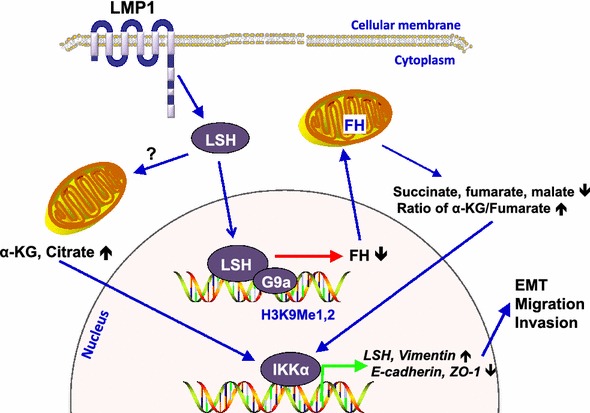
Schematic model of lymphocyte-specific helicase (LSH) in cancer progression. LSH promotes cell growth, migration, and invasion, which are characteristics of cancer progression. The effect of LSH is, in part, mediated by fumarate hydratase (FH), through the intact combination of LSH and euchromatic histone-lysine N-methyltransferase 2 (G9a). FH repression, in turn, leads to changes of tricarboxylic acid cycle (TCA) intermediates, including succinate, fumarate, and malate, and an increase in the ratio of α-ketoglutarate (α-KG) to fumarate. TCA intermediates promote migration, invasion, and epithelial–mesenchymal transition (EMT) through the decrease of E-cadherin and tight junction protein ZO-1 and the increase of vimentin. The changes of E-cadherin and ZO-1 are mediated by inhibitor of nuclear factor kappa-B kinase alpha (IKKα), which directly binds to these promoters
